# Optical Approaches for Investigating Neuromodulation and G Protein–Coupled Receptor Signaling

**DOI:** 10.1124/pharmrev.122.000584

**Published:** 2023-11

**Authors:** David J. Marcus, Michael R. Bruchas

**Affiliations:** Center for the Neurobiology of Addiction, Pain and Emotion (D.J.M., M.R.B.), Department of Anesthesiology and Pain Medicine (D.J.M., M.R.B.), Department of Pharmacology (M.R.B.), and Department of Bioengineering (M.R.B.), University of Washington, Seattle, Washington

## Abstract

Despite the fact that roughly 40% of all US Food and Drug Administration (FDA)-approved pharmacological therapeutics target G protein–coupled receptors (GPCRs), there remains a gap in our understanding of the physiologic and functional role of these receptors at the systems level. Although heterologous expression systems and in vitro assays have revealed a tremendous amount about GPCR signaling cascades, how these cascades interact across cell types, tissues, and organ systems remains obscure. Classic behavioral pharmacology experiments lack both the temporal and spatial resolution to resolve these long-standing issues. Over the past half century, there has been a concerted effort toward the development of optical tools for understanding GPCR signaling. From initial ligand uncaging approaches to more recent development of optogenetic techniques, these strategies have allowed researchers to probe longstanding questions in GPCR pharmacology both in vivo and in vitro. These tools have been employed across biologic systems and have allowed for interrogation of everything from specific intramolecular events to pharmacology at the systems level in a spatiotemporally specific manner. In this review, we present a historical perspective on the motivation behind and development of a variety of optical toolkits that have been generated to probe GPCR signaling. Here we highlight how these tools have been used in vivo to uncover the functional role of distinct populations of GPCRs and their signaling cascades at a systems level.

**Significance Statement:**

G protein–coupled receptors (GPCRs) remain one of the most targeted classes of proteins for pharmaceutical intervention, yet we still have a limited understanding of how their unique signaling cascades effect physiology and behavior at the systems level. In this review, we discuss a vast array of optical techniques that have been devised to probe GPCR signaling both in vitro and in vivo.

## I. Introduction

Throughout the history of research in neuroscience and psychology, the mechanisms underlying brain function have primarily been treated as a “black box.” Although inputs and outputs to and from the brain can be reliably tracked, how these networks ultimately map onto the function of the brain in behavior is often inferred through correlative studies. As in other domains of science, both theoretical and technological advancements have been necessary to expand and gain a deeper understanding of the brain within this limited conceptual framework.

A pivotal breakthrough in our understanding of the principals of neural signaling was the precise description of the electrically excitable nature of neurons, exquisitely detailed in a series of seminal manuscripts by Alan Hodgkin and Andrew Huxley (Hodgkin et al., 1952; Hodgkin and Huxley, 1952a,b). These pioneering ex vivo studies of the squid giant axon paved the way early attempts to record electrical activity within awake animal brains. Many of these initial studies sought to understand the neural correlates of perception, with particular emphasis placed on the processing of visual information in the striate cortex. David Hubel and Torsten Wiesel pioneered a number of novel experimental and computational approaches for understanding the visual coding properties of neurons in the primary visual cortex (Hubel and Wiesel, 1959).

However, a critical component from these experiments that limited our conceptual understanding was the establishment of causal links between neural activity and perception. For example, could one manipulate activity of an orientation-selective neuron to interfere with that specific aspect of visual perception while sparing the processing of color or contrast? The inability to manipulate the activity of functionally distinct neural populations was not unique to these studies. Indeed, this represented a significant methodological barrier that hindered neuroscience for several decades. Although lesions or intracranial electrical stimulation studies provided a gross understanding of the function of general brain regions, they offered no insight into the unique function of intermingled neural populations within these brain regions. This caveat was understood early on and explicitly stated in the 1979 article by Francis Crick entitled “Thinking about the Brain” (Crick, 1979). In this piece, he described the necessity for a technique that would allow “all neurons of just one type [to be] inactivated, leaving the others more or less unaltered.” In subsequent works, Crick made the conjecture that “harnessing the precision of light” could be adapted for this purpose, as it possesses suitable temporal and spatial properties needed to dissect the intricacies of neural signaling.

Although this speculation was not lost the neuroscientific community, the first demonstration of using light to control the activity of specific neural populations did not arrive to the field for more than two decades. A study by Zemelman et al. (2002) describes a novel technique known as “chARG,” which rendered vertebrate neurons sensitive to light. In a pivotal series of experiments, they demonstrated that by cotransfection of multiple components of the *Drosophila* photoreceptor system into cultured rat hippocampal neurons, visible light irradiation led to increased excitability and action potential firing.

This study represented a crucial step forward, but the technique was not widely adopted due to both the multicomponent transfection and the slower kinetics of this neuromodulatory approach via G protein–coupled receptor (GPCR)-based opsin. This precluded easy genetic targetability as well as the millisecond-scale temporal precision necessary to faithfully manipulate neural activity. In 2005, a study led by Ed Boyden and Karl Deisseroth described a novel optical toolkit that seemingly met both of these requirements. This system made use of a single component blue light–gated cation channel called channelrhodopsin 2 (ChR2), which is natively expressed in certain species of algae to drive phototaxis (Boyden et al., 2005). This manuscript demonstrated that expression of a genetically engineered variant of this molecule in mammalian tissues was sufficient to render neurons sensitive to blue light (450–490 nm), which drove robust action potential firing. The tractability of this “optogenetic” tool was independently characterized in several other laboratories, and it was swiftly adopted by researchers across the globe to answer many longstanding neuroscientific questions (Li et al., 2005; Nagel et al., 2005; Bi et al., 2006; Ishizuka et al., 2006). This approach has now been under constant tuning and numerous developments since its initial iteration. This has led to the implementation of dozens of new ion channel–based optogenetic tools that have unique temporal and spectral properties and allow for neuronal silencing as well as activation. The structure, biophysics, and implementation of these tools has been widely reviewed over the decade and will not be a primary focus of this review (Fenno et al., 2011; Deisseroth, 2015; Wiegert et al., 2017; Rost et al., 2022).

Although the ChR2-based optogenetic toolkit has been revolutionary for researchers to gain direct access to control the excitability of neurons, the brain does not function solely as a binary digital processor (on/off) of information flow. Indeed, the brain produces dozens of neuromodulatory gain signals which fine-tune, stabilize, and alter distinct neural circuits through G protein–coupled receptors (GPCRs). Direct actuation of ion channels would be insufficient to recapitulate the functional properties many of these neuromodulatory systems. GPCRs represent one of the most targeted protein families for pharmaceutical intervention, with roughly 40% of all US Food and Drug Administration (FDA)-approved drugs interacting with these receptors (Kenakin, 2002). Although there have been decades of biochemical, pharmacological, and structural studies of these receptors in in vitro heterologous expression systems, there has still been limited ability to examine the functional properties of how GPCRs modulate intact neural circuits to ultimately shape observable behaviors. To address these limitations, there has been a concerted effort to develop high-resolution approaches that allow for probing GPCR signaling with the same spatial and genetic specificity afforded by the initial optogenetic toolkit. In this review, we synthesize the vast array of unique optical approaches that have been implemented to gain a more complete understanding of GPCR signaling, and neuromodulatory processes in behavior. This review will focus solely on techniques to manipulate neuromodulatory signaling pathways, and will not include the development of optical approaches to record neural activity and neuromodulatory signals where other strong reviews cover this topic including: (Tian et al., 2012; Alivisatos et al., 2013; Dong et al., 2022; Tjahjono et al., 2022.

## II. Development of a GPCR-Based Optical Toolkit

The photoreceptive protein rhodopsin was discovered almost 150 years ago by the German scientist Franz Christian Boll (Marmor and Martin, 1978). Given the central role of this protein is human vision, it has been among the most highly studied proteins to date. Indeed, it was the first GPCR to have its crystal structure solved, and the entire class A family of GPCRs is denoted as “rhodopsin-like” (Palczewski et al., 2000), which are among the most highly used pharmaceutical targets (Rosenbaum et al., 2009; Yang et al., 2021). The study of the rhodopsin protein spurred the discovery of numerous photoreceptive proteins across all domains of biology. Although these necessarily share the common feature of being light sensitive, the cellular signaling, physiologic impact, and function of these proteins are quite distinct. The vast array of opsin subtypes across species and the unique spectral properties of these opsins offer tantalizing opportunities for scientists to harness variants of these proteins for the modulation of unique intracellular signaling cascades.

### A. Photoactivation Cycles of Light-Sensitive GPCRs

**Fig. 1:**
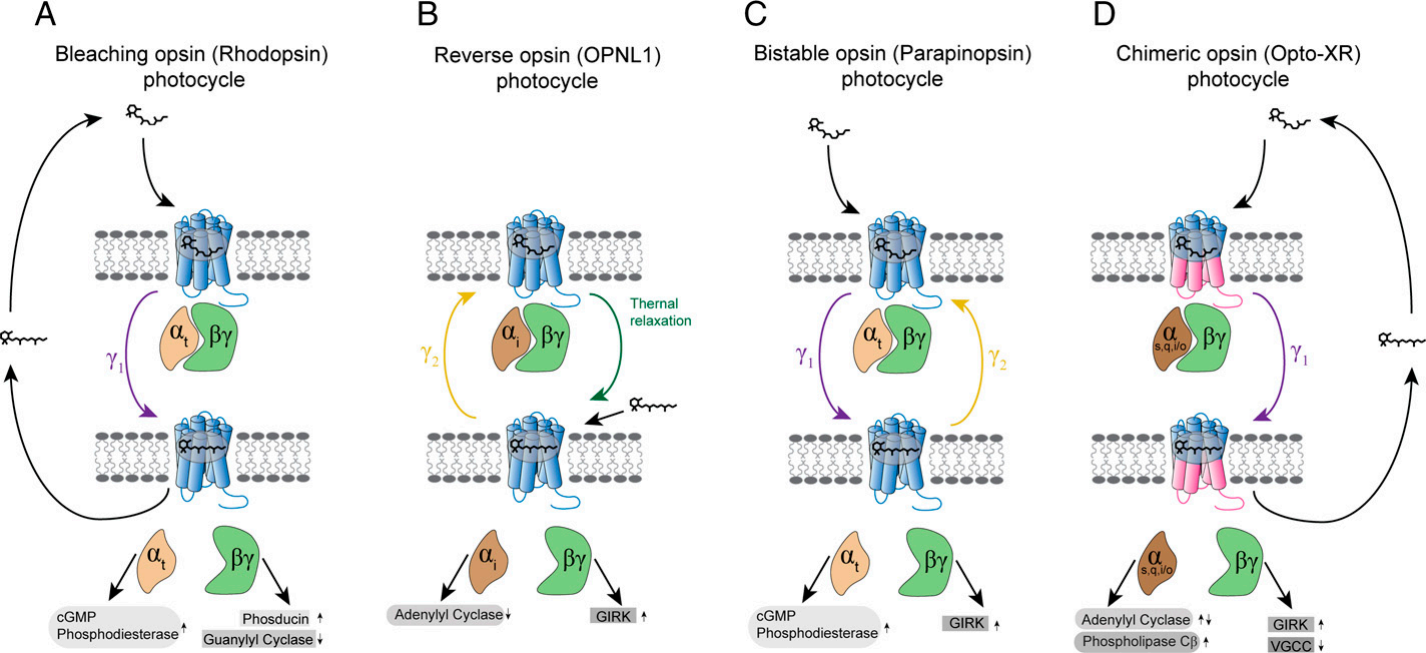
Photocycles of GPCR opsins. Schematic demonstrating photocycles for (A) bleaching, (C) reverse, (D) bistable, and (D) chimeric opsins. Cis-retinol bound receptors are in the “off state” in complex with their cognate G proteins. For bleaching opsins (in this case, rhodopsin), (A), after exposure to light, the receptor-bound cis-retinol undergoes cis-trans isomerization, leading to a conformational change in the receptor to its “on state.” This change in turn catalyzes the exchange of GDP for GTP in the cognate heterotrimeric G protein, leading to its dissociation from the receptor. The active G protein leads to increased activity of cGMP phosphodiesterase and phosducin through its *α* and *βγ* subunits, respectively, and decreased guanylyl cyclase through its *βγ* subunit. In order for the photocycle to reset, the bound trans-retinal must dissociate from the receptor and be replaced with cis-retinol. For reverse opsins (B), trans-retinol can directly bind and activate the receptor, acting much like a pharmacological agonist. Light irradiation in this case leads to trans-cis isomerization, driving an inactive receptor conformation. In the case of OPNL1, bound cis-retinol is capable of thermally relaxing to the trans configuration, leading to receptor activation. For bistable opsins (C), one wavelength of light (in the case of parapinopsin, UV) induces cis-trans isomerization of the receptor-bound retinol, leading to receptor activation. Irradiation with a different wavelength of light (in this case, amber) is capable of inducing trans-cis isomerization, reverting the receptor back to its inactive form without necessitating retinol dissociation. For an Opto-XR chimeric receptor (D), specific C-terminal residues of a rhodopsin molecule are swapped for C-terminal residues of a nonvisual GPCR of interest. Light irradiation induces cis-trans retinol isomerization, activating the receptor. The active G protein drives increased or decreased adenylyl cyclase activity through the *α* subunit, depending on the selected GPCR chimera, increased GIRK conductance and PLC*β* activity through the *βγ*, and decreased voltage-gated calcium channel (VGCC) conductance through the *βγ* subunit.

Similar to the aforementioned channelrhodopsins, the GPCR family of opsins binds the chromophore retinal, which undergoes light-dependent isomerization, imbuing these proteins light sensitivity (Maeda and Yoshizawa, 1982). The most highly studied of these opsins is the vertebrate rhodopsin, the primary opsin in the human retina, which is principally expressed in rod cells and mediates dim light vision (Litman and Mitchell, 1996; Shichida and Imai, 1998). Rhodopsin’s photocycle is characterized by strong photobleaching properties. In its “off” configuration, rhodopsin is bound by the cis isomer of the chromophore retinal, which becomes rapidly converted to trans after light exposure (Bownds, 1967; Bridges, 1986) (Fig. 1A). Light-induced isomerization results in a conformational state change in the opsin, ultimately activating the G_t_ protein signaling pathway. The now liberated G_t_ subunit subsequently activates cGMP phosphodiesterase, leading to decreased cGMP levels (Stryer et al., 1981). This eventually results in hyperpolarization of the photoreceptive neuron due to reduced conductance through cGMP-dependent cation channels (Ripps, 2001). The photobleaching properties of the rhodopsin protein are driven by the trans-retinal’s inability to become isomerized back toward cis-retinal and must thus fully dissociate from the opsin to be replaced by diffusing cis-retinal, ultimately restarting the photocycle (Deterre et al., 1987). Although the same general photocycle is preserved in cone opsins, which mediate color vision, these pigments typically display significantly shorter deactivation kinetics compared with rhodopsin (Nathans, 1999; Masseck et al., 2014; Berry et al., 2019).

A separate family of GPCR opsins, collectively referred to as bistable opsins, share similar photoactivation properties; however, they differ in the mechanism by which they return to the “off” state. One group of bistable opsins are the parapinopsins, which are found in certain species of catfish and lamprey (Terakita, 2005). Similar to rhodopsin, specific wavelengths of light, in this specific case UV, induce isomerization of the bound cis-retinal chromophore, leading to activation of downstream G protein signaling. However, unlike rhodopsin, parapinopsin is capable of reverse isomerization of trans-retinal back to cis-retinal (Koyanagi et al., 2004; Kawano-Yamashita et al., 2015). Specifically, after exposure to amber wavelength light, this trans-cis isomerization is catalyzed, leading to a reversion to the cis-bound off state of the opsin (Copits et al., 2021).

Interestingly, a recently characterized group of opsins displays the inverse photocycle. These opsins, such as the spider peropsin or chicken Opn5L1, are capable of directly binding diffuse trans-retinal in the dark, which activates the receptor and functions similarly to a classic GPCR agonist (Sato et al., 2018; Nagata et al., 2018; Yamashita, 2020). After exposure to light, the bound trans-retinal is isomerized to cis configuration, leading to chromophore dissociation and reversal to the “off” state.

### B. Native GPCR Opsins for Modulation of Neural Activity

Although initial optogenetic approaches using GPCR-based opsins proved too cumbersome to garner widespread adoption, the cloning and characterization of novel opsins has led to the implementation of numerous native light sensitive GPCRs for the control of neuromodulatory signaling (Kleinlogel, 2016). Some early studies using heterologous expression of vertebrate rhodopsin were able to demonstrate that this receptor could couple to G_i_ proteins in nonvisual cells and inhibit excitability, but these experiments suffered from undesirable signaling properties, namely slow deactivation kinetics and rapid response rundown due to photobleaching (Zemelman et al., 2002; Li et al., 2005). More recent studies have opted to use cone photoreceptors, such as the short- and long-wavelength opsins (vSWO and vLWO), due to their significantly improved reactivation kinetics and photobleaching properties (Kawamura and Tachibanaki, 2008; Masseck et al., 2014).

Over the past few decades, bistable opsins have been characterized as efficient modulators of neural activity (Kleinlogel, 2016). There are three known vertebrate bistable opsins: encephalopsin (OPN3), melanopsin (OPN4), and neuropsin (OPN5) (Provencio et al., 1998; Blackshaw and Snyder, 1999; Tarttelin et al., 2003). When expressed in neurons, both OPN3 and OPN5 couple to G_i_ signaling pathways, driving neuronal inhibition (Yamashita et al., 2010, p. 5). Interestingly, OPN3 (distinguished from the invertebrate eOPN3, discussed subsequently) is natively expressed in various nonvisual neuron populations in the brains of vertebrates and hence has not been widely used as an optogenetic tool (Koyanagi et al., 2013, p. 3). Unlike the vertebrate opsins that result in cellular hyperpolarization, melanopsin is a G_q_-coupled receptor, and as such its activation by blue light will result in inositol triphosphate (IP_3_) generation and subsequent calcium release and neuronal depolarization (Provencio et al., 1998). Heterologous expression of this receptor in neurons has been demonstrated by several groups to modulate behavioral responses in awake mice and rats (Melyan et al., 2005; Lin et al., 2008; Tsunematsu et al., 2013). A recent study demonstrated that optogenetic activation of this receptor expressed in hypothalamic orexin neurons was capable of controlling sleep and wake cycles in vivo (Tsunematsu et al., 2013).

More recent focus has shifted to the development and characterization of nonvertebrate opsins for decreasing neuronal excitability. Three research groups independently investigated the use of the lamprey parapinopsin (PPO) for efficient optogenetic silencing (Eickelbeck et al., 2020; Copits et al., 2021; Rodgers et al., 2021). Although multiple ion channel-based opsins have been developed for neuronal silencing, the majority have low efficacy or paradoxical excitatory effects when applied to presynaptic terminals. In the case of proton pump–derived optogenetic tools such as ArchT, cytosolic alkalinization as a result of proton efflux can result in Ca^2+^ influx, leading to increased spontaneous transmitter release (Mahn et al., 2016). Similarly, anion-conducting channelrhodopsins such as halorhodopsin can also lead to unexpected depolarization due to the elevated basal chloride concentrations in axon terminals [see Rost et al. (2022) for further discussion of optogenetics at the presynapse]. These caveats directly spurred the development of GPCR-based inhibitory tools such as PPO. Initial studies using PPO demonstrated that conversion to the active state required UV light, leading to concerns regarding its utility in vivo due to the cytotoxic effect of UV light. However, further investigation of this opsin demonstrated that its spectral range was broader than previously appreciated, namely that blue light was capable of efficiently activating the receptor, leading to inhibition of cyclic adenosine monophosphate (cAMP) accumulation and voltage-gated calcium channels, and activation of G protein–coupled inward rectifying potassium channels (GIRKs) (Copits et al., 2021). Notably, blue light is sufficient to drive inhibition of release in presynaptic terminals, both in vitro and in vivo. These studies further demonstrated the two-photon sensitivity of this opsin, leading to mCherry-tagged *γ*9 subunit translocation after photostimulation. In tandem with this finding, a parallel publication described the application of mosquito rhodopsin eOPN3, which similarly couples to G_i/o_ signaling pathways and results in efficient neuronal silencing in both axon terminals and somatodendritic compartments (Mahn et al., 2021). This study also demonstrated robust inhibition in hippocampal synapses, alongside in vivo utility by eliciting ipsiversive rotation in mice after inhibition of substantia nigra (SN) dopaminergic terminals in the dorsomedial striatum (DMS). Importantly, eOPN3 shows maximal activation from green light illumination, allowing this tool to be potentially multiplexed with other opsins or biosensors with spectral properties tuned to far-shifted blue or red light. Additional nonvertebrate opsins have since been characterized for efficient manipulations of neural activity, including the zebrafish Opn7b (Karapinar et al., 2021).

### C. Chimeric Opsin GPCRs

The development of optogenetic techniques was primarily driven by the desire to have genetically targetable spatiotemporal control of the excitability of specific neuronal populations. However, in tandem with the development of these tools, multiple groups harnessed the power of optogenetics to gain access to GPCR signaling. Interestingly, even prior to the development of channelrhodopsin-based optogenetic methods, work by Khorana and colleagues characterized a novel chimeric protein that they developed, a fusion of the vertebrate visual rhodopsin and the beta-2 adrenergic receptor (*β*_2_AR) (Kim et al., 2005). Specifically, they performed a series of mutagenesis experiments in which components of the rhodopsin intracellular loops and C terminus were swapped for the intracellular components of the *β*_2_AR. These loops contain critical phosphorylation and glycosylation sites, crucial for the trafficking of the receptor to specific subcellular compartments and for the receptor’s unique coupling properties to G proteins. After the selection of a suitable chimera that showed stable expression in the plasma membrane, they demonstrated that light activation of the receptor drove intracellular G_s_ signaling, which is typically recruited by native *β*_2_AR activation but not by rhodopsin activation.

Subsequent studies by Deisseroth and colleagues expanded on this study with the development of the “Opto-XR” toolkit (Airan et al., 2009) (Fig. 1B). These initial reports again focused on adrenergic receptor signaling, creating novel rhodopsin–alpha-1 adrenergic receptor (*α*_1_AR) and rhodopsin-*β*_2_AR receptor chimeras, demonstrating comparable levels of G protein recruitment to native receptors after light irradiation. Uniquely, this study illustrated for the first time the use of these chimeras in vivo. Not only did optical activation of the opto-*β*_2_AR alter innate neural excitability and firing properties, but it also drove robust conditioned place preference when it was expressed and activated on neurons in the shell of the nucleus accumbens (NAc). Other groups have recapitulated the in vivo viability of opto-*β*_2_AR, displaying its ability to drive anxiety-like behaviors and alter contextual encoding of fear memories (Siuda et al., 2015b, 2016; Seo et al., 2021; Lei et al., 2022).

Further refinements of the opto-*β*_2_AR have aimed to expand our understanding of the in vivo function of specific intracellular signaling cascades recruited during *β*_2_AR stimulation. In addition to signaling through canonical G protein pathways, most GPCRs recruit arrestin molecules to activate a complementary signaling pathway, typically though the recruitment of the mitogen-activated protein kinase (MAPK) family of proteins (Pierce and Lefkowitz, 2001). A study by Bruchas and colleagues took advantage of these divergent signaling pathways to design biased variants of the opto-*β*_2_AR that drove activation of either G protein signal transduction or arrestin signal transduction pathways (Siuda et al., 2015b). Through the mutation of key residues on the intracellular loops or C terminus of the opto-*β*_2_AR that were necessary for either G protein or arrestin recruitment, they were we able to make optogenetically activatable receptors that only recruited one of the two signal transduction pathways. These tools would allow for specific dissection of the functional role of these unique intracellular signaling pathways in vivo as well as in vitro.

Since the initial establishment of an Opto-XR toolkit based on *β*_2_AR, multiple groups have developed a range of optogenetically activatable GPCRs. Less than a year after the initial publication of the opto-*α*_1_AR and the opto-*β*_2_AR, a study by Herlitze and colleagues validated an optogenetically activatable variant of the serotonin receptor 1A (5-HT_1A_) (Oh et al., 2010). These experiments followed a similar mutagenesis approach, replacing the C terminus of the vertebrate rhodopsin with the C terminus of the 5-HT_1A_, leading to similar expression patterns to native 5-HT_1A_. This study was soon followed by the development of optogenetically activatable variants of other monoaminergic receptors, notably the 5-HT_2A_ receptor (Eickelbeck et al., 2019) and dopamine receptor 1 (D_1_R). Notably, the study that generated the opto-D_1_R showed that activation of this receptor on natively D_1_R neurons on the NAc could recapitulate the behavioral effects of dopamine terminal stimulation in this region (Gunaydin et al., 2014). This experimental design highlights the array of novel questions that can be answered using this technique. In a spatiotemporally specific manner, the authors causally linked the behavioral effects of presynaptic neuromodulator release with the activation of a specific subclass of GPCRs on a genetically defined cell population. The Opto-XR toolkit has since expanded to multiple unique GPCR subtypes, including chemokine (Xu et al., 2014), metabotropic glutamate (van Wyk et al., 2015), opioid (Barish et al., 2013; Siuda et al., 2015a; Castro et al., 2021), adenosine (Li et al., 2015), 5HT_2A_ (McGregor et al., 2016), and dozens of orphan GPCRs (Morri et al., 2018) (see Table 1 for further description of these tools).

**TABLE 1:** Chimeric receptors

Chimeric Receptor	Effect	Source
*β*_2_-AR	Increased cAMP Decreased network firing CPP when activated on NAc Shell neurons	(Kim et al., 2005) (Airan et al., 2009)
*α*_1_AR	Increased PLC activity Increased neural activity	(Airan et al., 2009)
5HT_1A_	Mimics native 5HT_1A_ expression pattern Neuronal hyperpolarization	(Oh et al., 2010)
*μ*-Opioid	Decreased calcium influx Decreased AC activity Increased GIRK conductance Decreased neuronal excitability Decreased sucrose consumption when activated on DRN terminals in the NAc	(Barish et al., 2013) (Siuda et al., 2015a) (Castro et al., 2021)
D_1_R	Increased cAMP Increased social preference when expressed on D_1_R+ NAc neurons	(Gunaydin et al., 2014)
*β*_2_-AR^LYY^ *β*_2_-AR^SS^	Arrestin- and G protein–biased *β*_2_-AR Increased in phosphor-ERK and cAMP, respectively	(Siuda et al., 2015b)
Adenosine_2A_	Increased cAMP, phospho-MAPK, and phosphor-CREB Increased spatial memory performance when activated on hippocampal neurons	(Li et al., 2015)
mGluR_6_	Increased GIRK currents Restoration of vision when expressed on ON-Bipolar retinal neurons	(van Wyk et al., 2015)
5HT_2A_	Neuronal hyperpolarization Mimics native 5HT_2A_ expression pattern	(McGregor et al., 2016) (Eickelbeck et al., 2019)
Orphan GPCRs	Varied	(Morri et al., 2018)

Despite these bioengineering advancements, there remain significant caveats to the implementation of these approaches. Chiefly among these concerns is the extent to which these chimeric opsins truly recapitulate the endogenous function of the receptor. Typically, these chimeric proteins are expressed under nonreceptor-specific promoters, leading to ectopic expression in non-native patterns. For example, if a chimeric D_2_R-rhodopsin chimera is expressed under the synapsin promoter, to what extend will optogenetic activation of this receptor truly recapitulate the endogenous function of the D_2_R? In this case, activation of this receptor may more closely model generalized G_i/o_ recruitment rather than a receptor-specific effect. These concerns can be partially ameliorated by specifically expressing the chimera under the control of the native receptor’s promoter or by inducing specific mutations to alter trafficking or subcellular localization of the receptor. Furthermore, it is well documented that rhodopsin displays strong photobleaching properties, bringing into question the in vivo utility of rhodopsin chimeras (e.g., can the receptor be repeatedly stimulated while maintaining its efficacy) (Deterre et al., 1987; Zemelman et al., 2002). This drawback could be addressed by generating a new series of chimeras that instead use opsins with more desirable photobleaching properties (e.g., lamprey parapinopsin) (Copits et al., 2021; Rodgers et al., 2021).

Despite advancements in bioengineering, there are significant caveats to implementing these approaches. The primary concern is the extent to which chimeric opsins recapitulate the endogenous function of the receptor. Typically, these proteins are expressed under nonreceptor specific promoters, leading to ectopic expression in non-native patterns. For example, if a chimeric D2R-rhodopsin chimera is expressed under the synapsin promoter, it may more closely model generalized G_i/o_ recruitment rather than a receptor-specific effect. To address this concern, chimeras can be specifically expressed under (Siuda et al., 2015b) the control of the native receptor’s promoter or by inducing specific mutations to alter trafficking or subcellular localization of the receptor. In two recent studies, authors compared the effects of stimulation of Opto-XRs (optoMOR, optoBeta2-AR, or optoBeta2-AR-mut) directly to their wild-type agonist-sensitive counterparts across numerous canonical signaling pathways at each receptor, including cAMP generation, kinetics of activation, MAPK activity, ion channel coupling, desensitization, internalization, and similarly in both cell lines, neurons and behavior. In particular, in optoMOR it was found that both endogenous MOR and opto-MOR couple to the same pools of beta-gamma that activate GIRK currents. Although these receptors are not the native receptor in many examples, they do indeed communicate with and activate endogenous signaling pathways in a similar manner to wild-type receptors.

Additionally, rhodopsin displays strong photobleaching properties, which brings into question the in vivo utility of rhodopsin chimeras. Can the receptor be repeatedly stimulated while maintaining its efficacy? To address this drawback, a new series of chimeras could use opsins with more desirable photobleaching properties, such as lamprey parapinopsin.

In summary, despite the advancements in bioengineering, significant concerns remain regarding the extent to which chimeric opsins recapitulate the endogenous function of the receptor. These concerns can be partially ameliorated by specific expression and mutation strategies. Further research into opsins with more desirable photobleaching properties may also improve the viability of chimeric opsins for in vivo use (Deterre et al., 1987; Zemelman et al., 2002; Copits et al., 2021; Rodgers et al., 2021).

## III. Photopharmacology for Spatiotemporal-Specific Control of GPCR Signaling

**Fig. 2:**
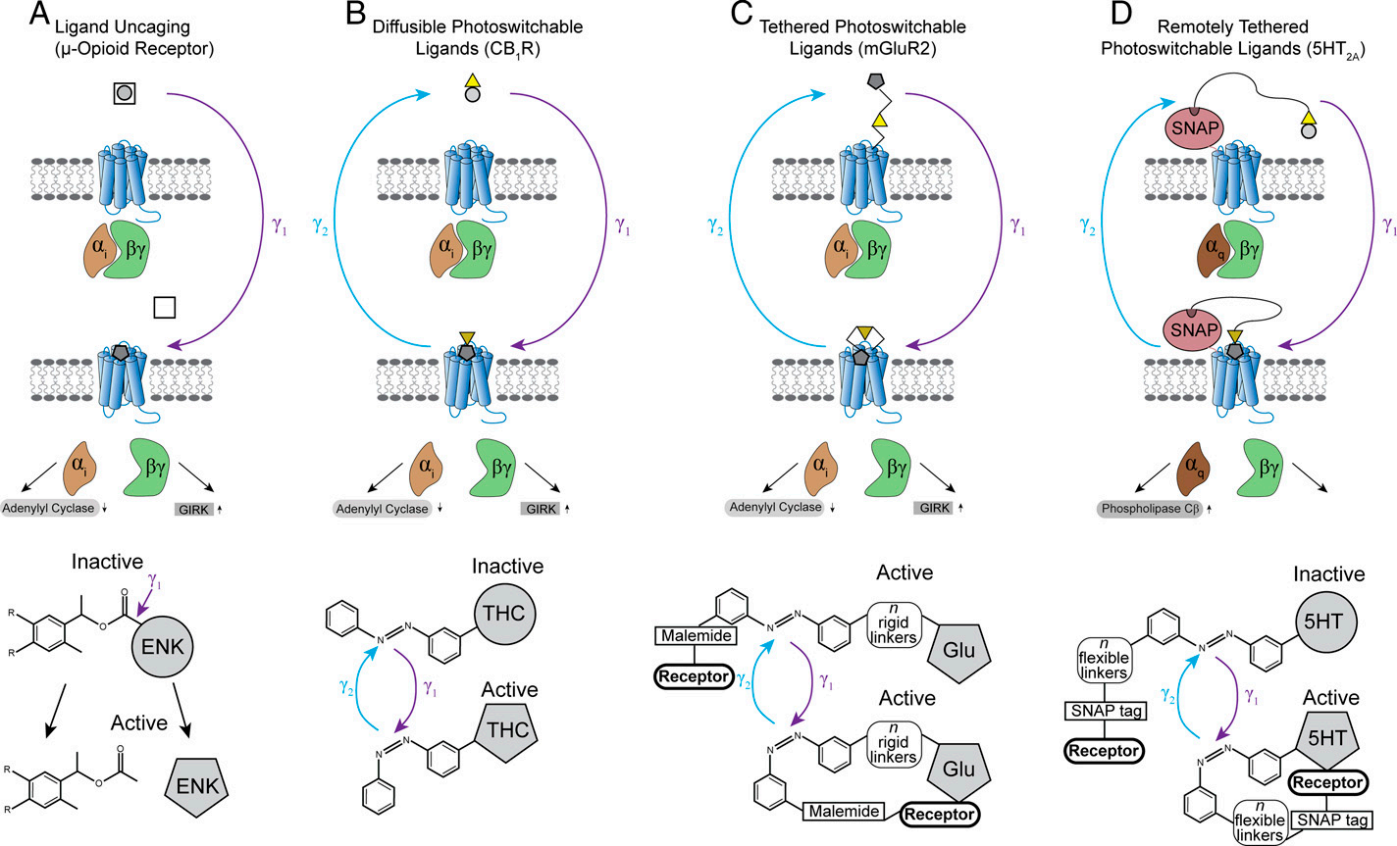
Photopharmacology. (A) Ligand uncaging makes use of a photolabile protecting group (e.g., nitrobenzyl) conjugated to the compound of interest. After light (typically UV) cleavage of this group, the compound (in this case, enkephalin) is irreversibly liberated and able to interact with its cognate receptor. (B) Photoswitchable ligands (in this case Δ^9^-THC) have a photoisomerizable moiety (e.g., azobenzene) conjugated to them in a manner that allows for light-dependent conversion from an inactive to active form. After irradiation with a specific wavelength of light (*γ*_1_), the photoswitchable group isomerizes (typically cis-trans) leading to an active form of the conjugated ligand, which can then bind to its cognate receptor. Irradiation with a different specific wavelength (*γ*_2_) reverses the isomerization and reverts the compound back to its inactive form. (C) Tethered photoswitchable ligands rely on a rigid linker that attaches the ligand (in this case glutamate) to the photoisomerizable group to specific (typically cysteine) residues on the receptor itself. After irradiation with a specific wavelength of light (*γ*_1_), the photoswitchable group isomerizes. In this case, the ligand is already in its active form, and the isomerization serves to either bring the ligand closer to the binding site or restrict it from the binding site. Note that the size and conformation of the linker are critically important in determining how the isomerization will ultimately effect ligand binding to the receptor. As with diffusible photoswitchable ligands, irradiation with a different specific wavelength (*γ*_2_) reverses the isomerization. (D) Orthogonally tethered photoswitchable ligands (in this case 5-HT) are directly conjugated to the photoisomerizable moiety, similar to diffusible photoswitchable ligands. However, this photoisomerizable group is attached via a flexible linker that binds to a self-labeling tag (e.g., SNAP), which itself is conjugated to the receptor of interest, far from the orthosteric site. After irradiation with a specific wavelength of light (*γ*_1_), the photoswitchable group isomerizes, leading to an active form of the conjugated ligand, which can then bind to its cognate receptor. Irradiation with a different specific wavelength (*γ*_2_) reverses the isomerization and reverts the compound back to its inactive form.

Although the use of chimeric opsins has allowed for a vastly increased ability to probe both the physiologic and behavioral effects of GPCR activation, they come with the caveat that they cannot precisely mimic the endogenous pattern of activation via ligand binding. Gaining a deeper insight into the innate function properties of native GPCRs necessitates the ability to directly manipulate ligand binding to specific receptor populations in a temporally specific manner. A powerful complementary approach that has been developed termed photopharmacology comprises a host of methods used to target light-regulated small molecules to native GPCRs (Fig. 2; Table 2). These techniques allow for temporally specific control of agonists, antagonists, or modulators of GPCR signaling both in vitro and in vivo.

**TABLE 2:** Photopharmacology

Type	Receptor/Effect	Source
Caged	Glutamate/agonist	(Callaway and Katz, 1993) (Ellis-Davies, 2007)
Caged	Adrenergic/agonists	(Muralidharan and Nerbonne, 1995)
Caged	GABA/agonist	(Gee et al., 1994)
Caged	*μ*- and *λ*-opioid/agonists	(Banghart and Sabatini, 2012) (Banghart et al., 2018)
Caged	D_1_R and D_2_R/agonists and antagonists	(Araya et al., 2013) (Gienger et al., 2020)
Caged	mGluR_5_/NAM	(Font et al., 2017)
Diffusible photoswitchable	mAChR M_1-5_/antagonist	(Nargeot et al., 1982)
Diffusible photoswitchable	mGluR_5_/NAM	(Pittolo et al., 2014)
Diffusible photoswitchable	A_2A_/partial agonist	(Bahamonde et al., 2014)
Diffusible photoswitchable	*μ*-opioid/agonist	(Schönberger and Trauner, 2014)
Diffusible photoswitchable	mGluR_5_/NAM	(Pittolo et al., 2014)
Diffusible photoswitchable	GLP-1/agonist	(Broichhagen et al., 2015b, 2016)
Diffusible photoswitchable	mGluR_4_/NAM and PAM	(Rovira et al., 2016)
Diffusible photoswitchable	mAChR M_1_/agonist	(Agnetta et al., 2017)
Diffusible photoswitchable	GPR40/ agonist	(Frank et al., 2017)
Diffusible photoswitchable	D_1_R and D_2_R/agonist	(Lachmann et al., 2017)
Diffusible photoswitchable	CB1/agonist	(Westphal et al., 2017)
Diffusible photoswitchable	CXCR3/agonist and antagonist	(Gómez-Santacana et al., 2018, 2019)
Diffusible photoswitchable	Histamine H_3_/agonist	(Hauwert et al., 2018)
Diffusible photoswitchable	CB2/agonist	(Sarott et al., 2021)
Diffusible photoswitchable	mGluR_2_/PAM	(Donthamsetti et al., 2021a)
Diffusible photoswitchable	5HT_2A_/agonist	(Gerwe et al., 2022)
Tethered photoswitchable	mAChR/agonist	(Lester et al., 1980) (Chabala and Lester, 1986)
Tethered photoswitchable	Shaker K^+^/antagonist	(Chambers et al., 2006)
Tethered photoswitchable	nAChR/agonist and antagonist	(Tochitsky et al., 2012) (Damijonaitis et al., 2015)
Tethered photoswitchable	mGluR_2_/agonist and antagonist	(Levitz et al., 2013)
Tethered photoswitchable	D_1_R and D_2_R/agonist	(Donthamsetti et al., 2017)
Remotely tethered photoswitchable	mGluR_2_/agonist	(Broichhagen et al., 2015a).
Remotely tethered photoswitchable	mGluR_6–8_/agonist	(Levitz et al., 2017)
Remotely tethered photoswitchable (nanobody)	mGluR_2_/agonist	(Farrants et al., 2018)
Remotely tethered photoswitchable	mGluR_2_/agonist	(Acosta-Ruiz et al., 2020) (Gutzeit et al., 2021)
Remotely tethered photoswitchable	GPR55/agonist	(Tobias et al., 2021)
Remotely tethered photoswitchable	D_1_R /agonist	(Donthamsetti et al., 2021b)
Remotely tethered photoswitchable	5HT_2A_/agonist	(Morstein et al., 2022)

### A. Ligand Uncaging

The first examples of photopharmacology approaches occurred decades before the advent of optogenetics. The ability of specific wavelengths of light to alter or break chemical bonds had been well documented since the early 1900s. This led to the idea that light might be used to photoconvert an inert compound into a biologically active one. One of the first demonstrations of this approach in biologic systems came in a study by Kaplan and colleagues (1978). Here, they used a photolabile nitrobenzyl group conjugated to the terminal phosphate of adenosine triphosphate (ATP), rendering it biologically inert, a form they denoted as “caged” (Kaplan et al., 1978) (Fig. 2A). After illumination with UV light, this nitrobenzyl group was cleaved, liberating (“uncaging”) the ATP molecule. This effect was assayed using a purified renal sodium-potassium ATPase. They demonstrated that only after photolysis of the nitrobenzyl group was the ATP able to be hydrolyzed by the ATPase. In the years since this publication, substantial work has gone into the development of novel photoremovable protecting groups that exhibit better spectral or kinetic properties. These include benzoin esters (Sheehan et al., 1971), 3-nitrophenyl esters (Kirby and Varvoglis, 1967; Harootunian et al., 1988), and methoxyphenacyl groups (Sheehan and Umezawa, 1973).

Although photouncaging techniques have since been applied to inorganic compounds, ions (Ellis-Davies, 2007), and even macromolecules such as G-actin (Marriott, 1994), primarily efforts have been placed toward the generation of photouncageable ligands for neurotransmitter receptors. Multiple small-molecule photouncageable ligands have been developed, including glutamate (Callaway and Katz, 1993; Ellis-Davies, 2007), GABA (Gee et al., 1994), a metabotropic glutamate receptor (mGluR)_5_ negative allosteric modulator (NAM) (Font et al., 2017), adrenergic receptor agonists (Muralidharan and Nerbonne, 1995), and dopamine receptor agonists and antagonists (Araya et al., 2013; Gienger et al., 2020). Additionally, recent developments have demonstrated the feasibility of photouncageable peptidergic signaling (Banghart and Sabatini, 2012; Banghart et al., 2018). A study by Banghart and Sabatini (2012) developed caged derivatives of the *μ*- and *λ*- receptor preferring opioid peptide Leu-enkephalin and the *κ*-receptor–preferring opioid peptide dynorphin. They demonstrated that UV uncaging of these compounds was able to drive activation of the *μ*-opioid receptor and *κ*-opioid receptor, respectively. Furthering this work, a subsequent study by this group developed a caged derivative of the opioid receptor antagonist naloxone. In addition to demonstrating its ability to block the *μ*-opioid receptor in vitro, they showed that it could attenuate agonist-induced ionic currents in ex vivo recordings from the locus coeruleus (Banghart and Sabatini, 2012).

Ligand uncaging has also been used in vivo to control animal behavior. A recent study by Ciruela and colleagues developed a caged version of the mGluR_5_ NAM raseglurant. This compound, JF-NP-26, exhibited similar pharmacological properties to the native compound after exposure to UV light. In preclinical models, raseglurant was capable of inducing analgesia in a model of neuropathic pain, but the locus of action in the brain for this effect remained unresolved. The authors first demonstrated that systemic injection of the caged compound JF-NP-26 had no effect in any pain assay. Then, after UV uncaging of this compound in the ventrobasal thalamus, a critical somatosensory relay center, they elicited robust antinociception comparable to that of systemically administered raseglurant. This study illustrates the power of photopharmacological techniques for determining the precise locus of action of pharmacological modulators of GPCR signaling (Font et al., 2017).

A caveat to ligand uncaging approaches is their lack of genetic targetability. In an intact system such as the brain, photoactivation of an uncageable compound leads to ligand binding to all receptors in the local vicinity, which can obscure specific effects on cell types or receptor populations of interest. This issue is compounded by the intrinsic nonspecificity of pharmacological agents. Additionally, these approaches are inherently irreversible. Although the onset of pharmacological action is rapid, the deactivation kinetics can be slower and limited by the diffusion or hydrolysis of the active compound.

### B. Photoswitchable Molecules

To overcome these temporal limitations, the last 15 years of research has seen the emergence of photoswitchable ligands (Ricart-Ortega et al., 2019). Most commonly, these compounds are built around a light-sensitive azobenzene or stilbene moiety that is capable of switching between cis and trans isomers (Ciccone and Halpern, 1959; Bortolus and Monti, 1979). Under dark conditions, azobenzenes exist primarily in a thermally stable trans configuration. After exposure to UV light, the molecule rapidly isomerizes from trans to cis. These compounds have garnered widespread usage in part due to the fact that the cis isomer is capable of converting back to the trans isomer via two different mechanisms. Firstly, simply removing the light irradiation will result in back conversion to the trans configuration via thermal isomerization (Beharry and Woolley, 2011). Interestingly, studies have demonstrated that as the wavelength of maximal absorption red shifts (i.e., longer wavelengths of light), the thermal relaxation (cis-trans) kinetics of the photoswitch generally tend to become more rapid (Pozhidaeva et al., 2004; Chi et al., 2006; Kamei et al., 2007; Beharry et al., 2008). However, the cis configuration also displays a unique spectral sensitivity, allowing for blue light isomerization back to the trans configuration should thermal isomerization prove to be too slow for the particular experimental question (Dhammika Bandara and Burdette, 2012; Merino and Ribagorda, 2012).

Photoisomerizable groups represent one class of chemical moieties used for photopharmacology, but subsequent studies have led to the development of novel methods for photoconversion of ligands from inactive to active states. A separate class of compounds such as the spiropyrans, diarylethene, and fulgides rely on light-induced switching from an open to a closed configuration (Berizzi and Goudet, 2020). The inactive state of these compounds is characterized by an open aromatic ring, which can be photoconverted to a closed state with UV light (Ricart-Ortega et al., 2019). Similar to the azobenzenes, these compounds can be back converted into their open state using visible light.

However, it should be noted that these photoswitchable compounds do not literally partition into “on” and “off” states. Indeed, the cis and trans probability states of these compounds often result in affinity changes of only a few fold (Hüll et al., 2018; Wijtmans et al., 2022). Compounding this issue is that both isomers of these photoswitchable ligands are based on the same design templates, making full efficacy shifts more challenging to accomplish. This necessitates careful consideration of ligand concentration. Often affinity and efficacy differences between these states go unreported, with many studies relying on only physiologic changes induced by photoisomerization (Wijtmans et al., 2022). This is not without exception, however, as recent studies have very rigorously demonstrated photoisomerization-induced efficacy shifts at the adenosine 2A (A_2A_) (Bahamonde et al., 2014), chemokine receptor 3 (CXCR3), (Gómez-Santacana et al., 2018, 2019), and 5HT_2A_ (Gerwe et al., 2022).

### C. Diffusible Photoswitchable Ligands

One of the predominant tools in photopharmacology is the diffusible photoswitchable ligand. Here, a compound is covalently linked to a photoswitchable group in such a way that light isomerization of that particular moiety (in the case of azobenzenes) converts the compound from an inactive to active form. These modified compounds are then either perfused over the sample in in vitro experiments or directly injected into the animal for in vivo experiments (Fig. 2B). The development of these tools began in the early 1980s, first using the compound 3,3′-bis-[*α*-(trimethylammonium)methyl] azobenzene (Bis-Q). Work by Erlanger and colleagues demonstrated that the native trans configuration of this compound functioned as a competitive antagonist at the muscarinic acetylcholine receptor (mAChR) (Bartels et al., 1971). However, after photoconversion of this compound to the trans isomer using UV light, the compound displayed an ∼3-fold reduction in potency at blocking the mAChR (Nargeot et al., 1982). Although these light-induced changes in potency are orders of magnitude smaller than those displayed by the current generation of photoswitchable ligands, they provide a crucial demonstration of the use of azobenzene moieties as photoswitches.

Much subsequent development of photoswitchable ligands has been focused on selective modulators of GPCR action. Recent advances in chemical synthesis have allowed for the creation of novel photoswitchable compounds based on highly lipophilic molecules such as the cannabinoid receptor 1 (CB1) agonist Δ-9 tetrahydrocannabinol (THC), the principal psychoactive constituent of marijuana. Frank and colleagues synthesized two unique azobenzene-conjugated THC derivatives that displayed high efficacy at the CB1 receptor in either the cis (Azo-THC-3) or trans (Azo-THC-4) configuration, respectively (Westphal et al., 2017). In this way, these compounds could be used to increase or decrease activation of the CB1 receptor in a temporally specific manner. Optical tools for investigating cannabinoid receptor 2 (CB2) have also been developed through the synthesis of a photoswitchable CB2 agonist called HU308 (Sarott et al., 2021).

These technologies have been expanded upon by generating photoswitchable compounds that display opposing activity at a receptor depending on the configuration of the conjugated photoswitchable moiety. Leurs and colleagues probed chemokine receptor 3 (CXCR3) function by creating a compound that could be photoconverted from an antagonist to an agonist. In the dark, the trans configuration of the compound they synthesized, VF16216, was capable of antagonizing CXCR3 in vitro (Gómez-Santacana et al., 2018, 2019). After stimulation with UV light, the azobenzene moiety photoconverted to the cis configuration, imbuing the compound with agonist-like effects at the same receptor. The compound was further able to be back converted to its trans isomer using blue light irradiation. These photoswitchable technologies have been expanded to produce novel ligands for a myriad of GPCRs, including an mGluR_4_ NAM (Rovira et al., 2016), mGluR_5_ (Pittolo et al., 2014), glucagon-like peptide 1 (GLP-1) (Broichhagen et al., 2015b, 2016), histamine H_3_ (Hauwert et al., 2018), muscarinic acetylcholine M_1_ (Agnetta et al., 2017), adenosine A_2A_ (Bahamonde et al., 2014), D_1_R and D_2_R (Donthamsetti et al., 2017; Lachmann et al., 2017), *μ*-opioid receptors (Schönberger and Trauner, 2014), GPR40 (Frank et al., 2017), 5HT_2A_ (Gerwe et al., 2022), and an mGluR_2_ positive allosteric modulator (Donthamsetti et al., 2021a) (see Table 2 for further information and “Barriers to Address” for further discussion of caveats to these approaches).

### D. Tethered Photoswitchable Ligands

A principal drawback to the use of diffusible photoswitchable ligands is the lack of genetic or cell type specificity, which can be readily achieved through modern optogenetic approaches. Recognizing this caveat, researchers have devised new methods to spatially or genetically restrict photoswitchable ligands to specific pools of the target protein of interest (Leippe et al., 2017). Pioneering experiments in the early 1980s found that reduction of disulfide bonds using dithiothreitol allowed for covalent linkage of trans-3- (*α*-bromomethyl)-3′- [*α*-(trimethylammonium) methyl]azobenzene (trans-QBr) to the extracellular moieties of receptors. QBr had previously been demonstrated to be a potent photoswitchable cholinergic agonist. Consistent with previous reports, the group demonstrated that light-induced photoisomerization was capable of increasing the potency of QBr several fold (Lester et al., 1980; Chabala and Lester, 1986). This tool represented a noted improvement over its predecessor, as receptor modulation was governed solely by intramolecular events rather than being diffusion limited as in the case of diffusible photoswitchable agonists. These experiments provided a proof of concept for a multitude of subsequent studies making the use of tethered photoswitchable ligands (Abreu and Levitz, 2020) (Fig. 2C).

The discovery that ligands could be covalently linked to extracellular cysteine residues proximal to the orthosteric binding site led to the first generation of genetically targetable photoswitchable tethered ligands. Studies by Trauner, Kramer, and colleagues first demonstrated these techniques using site-specific mutagenesis of shaker K^+^ channel to create a genetically modified channel that had ectopic expression of a cysteine residue in proximity to the agonist binding site (Chambers et al., 2006). They used a tripartite photoswitchable antagonist that contained a maleimide group for covalent conjugation to the cysteine residue, an azobenzene group for photoisomerization, and a tetraethylammonium group for blockage of the potassium channel pore. This compound, MAZ-AZO-QA, displayed potent blockade of the potassium channel conductance in the dark. However, after UV-induced trans-cis isomerization, the linker became too short to reach the potassium channel pore, leading to a reduction in receptor antagonism.

Subsequent studies by the same group have demonstrated the feasibility of this approach for the light-dependent regulation of GPCR signaling. A paper by Levitz and colleagues (2013) sought to develop an optical toolkit for spatiotemporal-specific control of mGluR2 signaling. Using an array of in silico and in vitro approaches, they performed site-directed mutagenesis on a number of amino acid residues proximal to the glutamate binding pocket (Levitz et al., 2013). In tandem, they generated photoswitchable tethered ligands to either antagonize (DMAG-1) or agonize (DMAG-0) mGluR_2_. As mGluR_2_ is G_i/o_ coupled, they found that UV irradiation decreased glutamate-evoked GIRK currents in the case of DMAG-1 and increased GIRK currents in the case of DMAG-0, effects that were reversible by irradiation with 500 nm light. They further showed that these tools could be used to modulate mGluR_3_ and mGluR_6_ as well as neuronal excitability in whole-cell patch clamp recordings in ex vivo brain slices and could modulate behavioral responses in vivo. They drove expression of the modified mGluR_2_ (LimGluR_2_) in larval zebrafish and assayed for the acoustic startle response (ASR). First, they demonstrated that pharmacological activation of native mGluR_2_s was sufficient to potentiate the ASR. Then, using LimGluR_2_, they showed bidirectional control over the ASR by activation or inhibition of the receptor using their photoswitchable tethered ligands. These pioneering studies have spurred the development of various photoswitchable tethered ligands for a number of GPCRs alongside nAChRs (Tochitsky et al., 2012; Damijonaitis et al., 2015), D_1_R, and D_2_R (Donthamsetti et al., 2017).

### E. Remotely Tethered Photoswitchable Ligands

Despite these advancements, there are numerous reactive extracellular cysteine residues, dramatically limiting the specificity of tethered ligand techniques. Secondly, mutation of these residues may have unintended consequences relating to expression, stability, or function of these receptors. Lastly, the maleimide group is unstable in aqueous environments, limiting its utility. To circumvent these limitations, many groups have developed the remotely tethered photoswitchable ligand toolkit. In this technique, a self-labeling tag (SNAP, HALO, CLIP, etc.) is conjugated to the receptor far from the orthosteric site (Keppler et al., 2003; Gautier et al., 2008; Los et al., 2008; Broichhagen and Levitz, 2022) (Fig. 2D). The tags are roughly the size of a fluorophore such as GFP and generally (albeit they can) do not lead to large-scale changes in receptor function. These chimeric proteins can be expressed under the control of a variety of promoters, leading to cell type specificity. The receptors bind moieties on engineered ligands that contain a long polyethylene glycol chain linked to a photoactivatable drug of choice, allowing for receptor modulation far from the compound’s binding site on the SNAP or HALO tag. The Trauner group has also been a leader in the development of these approaches. In a 2015 study examining mGluR_2_ function, they developed a remote tethering strategy that could lead to optical activation of the receptor both in vitro and in vivo with cell type specificity (Broichhagen et al., 2015a). By directly activating this chimeric receptor on PFC neurons that naturally express mGluR_2_, they were able to impair working memory, which had previously been demonstrated to be an effect of global mGluR_2_ agonism (Acosta-Ruiz et al., 2020; Gutzeit et al., 2021). Thus, they were able to harness this approach to find a specific neural site of action for these global effects. These strategies have since been extended to explore other GPCRs such as mGluR_6-8_ (Levitz et al., 2017)_,_ GPR55 (Tobias et al., 2021), and 5HT_2A_ (Morstein et al., 2022).

## IV. Optical Control of Intracellular Signaling Cascades

**Fig. 3:**
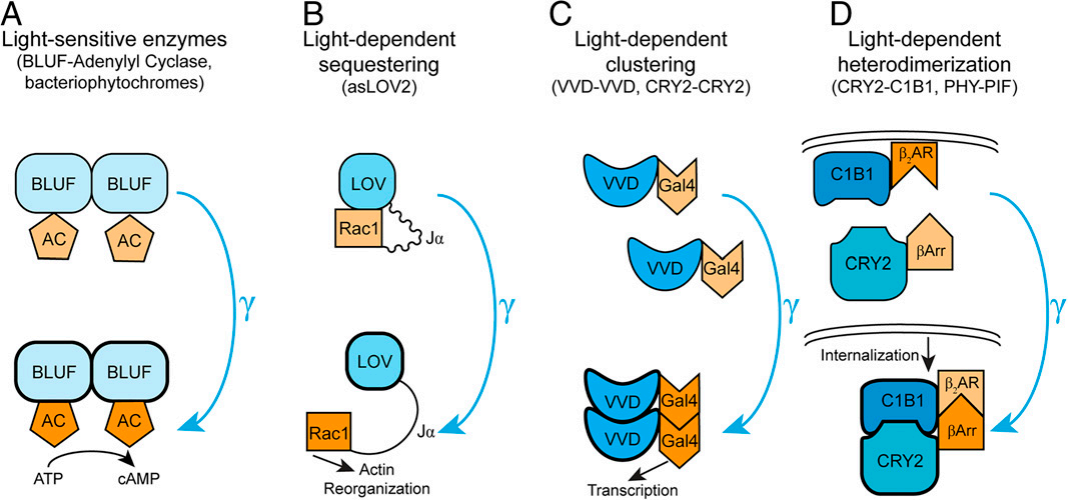
Optical control of intracellular signaling cascades. (A) Light-reactive protein domains such as BLUF undergo a conformational change when exposed to blue light. In nature, these domains are often directly conjugated to enzymes, such as adenylyl cyclase, such that the light-induced conformational change in the BLUF domain activates the associated enzyme (in this case, adenylyl cyclase). (B) AsLOV2 domains are a common strategy for light-dependent control of proteins or enzymes of interest. When fused to the LOV domain via the J*α* helix, the protein is sequestered proximally to the LOV domain, sterically inhibiting it from interacting with other components of its signal transduction cascade. After light-dependent unwinding of the J*α* helix, the now flexible linker allows the protein (in this case, Rac1) to diffuse away from LOV domain to interact with other protein partners (e.g., actin). (C) Many proteins, such as the RTKs or transcription factors, form functional homooligomers when activated. Light-dependent clustering schemes, such as those using the fungal photoreceptor VVD, have been used to gain optogenetic control over homooligomer formation (in this case, the transcription factor Gal4). (D) Alternatively, many studies have used light-evoked heterodimerization tools such as CRY2-C1B1 to gain control over specific protein-protein interactions. A light-induced conformational change in the CRY2 domain allows it to bind to its partner C1B1 and in doing so allows for conjugated proteins of interest to be brought in close proximity to each other (in this case, *β*-arrestin and the *β*_2_AR).

The aforementioned tools have allowed unprecedented ability to manipulate GPCRs with genetic, pharmacological, and temporal specificity (Ricart-Ortega et al., 2019). However, these methods are all primarily based around manipulating receptors rather than the cascade of signaling molecules downstream of receptor activation. For optogenetic tools based around ion channels, simple control over the opening or closing of the channel pore could be sufficient to recapitulate the general function of native ion channels. However, GPCR activation can lead to the recruitment of a myriad of intracellular effectors. Therefore, direct spatiotemporal control over these effector pathways could be incredibly informative for understanding the function of a GPCR signaling pathway in vitro and in vivo. Research over the last decade has seen substantial investment into the development of optical techniques that provide spatiotemporal access to intracellular signaling cascades (Fig. 3; Table 3).

**TABLE 3:** Optical control of intracellular signaling cascades

Targeted Signaling Molecule Family	Effect	Source
Heterotrimeric G protein	Inhibition of G*βγ* translocation via RGS4 activation	(O’Neill and Gautam, 2014)
Heterotrimeric G protein	Activation of G_q_ and G_s_ via recruitment to plasma membrane	(Yu et al., 2016)
Heterotrimeric G protein	Inhibition of G_q_ signal transduction via RGS2 activation	(Hannanta-Anan and Chow, 2018)
Heterotrimeric G protein	Activation of heterotrimeric G proteins	(Garcia-Marcos et al., 2020)
Arrestin	Arrestin recruitment to the plasma membrane and internalization of *β*_2_-AR	(Takenouchi et al., 2018)
Adenylyl cyclase	Increased currents through cAMP gated ion channels and modulation of drosophila grooming behavior	(Stierl et al., 2011)
Adenylyl cyclase	Increased cAMP production	(Scheib et al., 2018)
Guanylyl cyclase	Increase cGMP production	(Scheib et al., 2018)
Phosphodiesterase	Decrease in cAMP and cGMP levels	(Gasser et al., 2014)
Phosphodiesterase	Decrease in cAMP and cGMP levels	(Stabel et al., 2019)
Diacylglycerol	Increased TRP channel currents	(Leinders-Zufall et al., 2018)
PI_3_K	Increased PIP_3_ levels	(Kakumoto and Nakata, 2013)
GTPase	Increased Rho activity via GEF translocation to cell membrane	(Levskaya et al., 2009)
MAPK	Increased JNK and p38 MAPK activity	(Melero-Fernandez de Mera et al., 2017)
MAPK	Increased MEK1 activity	(Zhou et al., 2017)
Gal4	Increased transcription	(Nagasaki et al., 2022)

### A. Light-Reactive Protein Domains

In order for optical control of second messenger cascades to be possible, individual signaling components must be imbued with light sensitivity. The majority of the tools developed to accomplish this goal use naturally occurring light-sensitive proteins from plants and bacteria. One of the most commonly used is the blue light–using flavin adenine dinucleotide (BLUF) domain (Anderson et al., 2005; Christie et al., 2012). Flavoproteins are a diverse set of proteins found across domains of life that play essential roles in a variety of biologic processes. In both prokaryotes and eukaryotes, flavoproteins are characterized by enzymatic activity centered around a flavin adenine dinucleotide (FAD) or flavin mononucleotide (FMN) cofactor (Gauden et al., 2006). Pioneering basic science investigations of the unicellular flagellate *Euglena gracilis* characterized a unique dimeric light-sensitive adenylyl cyclase that was imbued with light sensitivity via its conjugation to a BLUF domain (Iseki et al., 2002) (Fig. 3A). The power of this tool for manipulating cAMP was used in a subsequent study by Nagel and colleagues, demonstrating light-gated adenylyl cyclase activity. After their in vitro characterization of the tool, they demonstrated profound in vivo reduction in grooming behavior in *Drosophila* expressing this construct following blue light illumination. (Schröder-Lang et al., 2007). As G_s_-coupled receptor activation similarly leads to increased production of cAMP, this tool has been used to optogenetically simulate the consequences of activating this arm of the GPCR effector pathway.

Light-oxygen voltage (LOV) domains represent another subclass of flavoproteins widely used for optogenetic control of second messenger cascades (Huala et al., 1997; Christie et al., 1998, 1999). LOV domains carry with them the added benefit of having two unique mechanisms that can be used for tool development: conformational change and oligomerization. The AsLOV2 domain from *Avena sativa* has been widely used to optogenetically manipulate protein-protein interactions (Harper et al., 2003; Yao et al., 2008). A recent study generated a constitutively active Rac1 mutant fused to the C terminus of AsLOV2, enabling photoswitchable control over Rac1 signaling. In the dark state, steric block prevented Rac1 activity by occluding it from downstream effectors (Wu et al., 2009, p. 1). Blue light irradiation elicited a conformational change in the fusion, unwinding the linking J*α* helix between Rac1 and AsLOV2, which resulted in localized Rac1 activation (Fig. 3B). This platform has also been used to study the effect of optogenetic control over inhibitory intracellular peptides that modulate endogenous kinase activity. A LOV-based oligomerization scheme has also been used to manipulate protein-protein interactions through the fungal photoreceptor Vivid (VVD) LOV domains, which are capable of forming homomers after light irradiation (Zoltowski et al., 2007; Zoltowski and Crane, 2008) (Fig. 3C).

A similar mechanism is shared with cryptochromes, another class of flavoproteins that have been used to study protein-protein interactions. This strategy primarily makes use of the cryptochrome CRY2 from *Arabidopsis thaliana* and its binding partner C1B1 (or C1BN) (Liu et al., 2008; Kennedy et al., 2010) (Fig. 3D). In the dark, these two components are typically found unassociated. However, light-induced conformational changes in their structure allows for the formation of CRY2-C1B1 heterodimers. The CRY2-C1B1 system has been used to manipulate G protein recruitment as well as to regulate G protein signaling (RGS) proteins and arrestin recruitment (see below). In addition to forming CRY2-C1B1 heterodimers, CRY2 is capable of forming light-induced homooligomers in the absence of C1B1 (Bugaj et al., 2013). This feature has been taken advantage of to understand the natural mechanism of receptor clustering, which is an important mechanism for the activation of many transmembrane receptors such as receptor tyrosine kinases (RTK) and many immune receptors. For example, fusion of the N-terminal Src-homology 2 (SH2) domain to CRY2 was able to drive light-induced clustering and activation of RTKs in vitro (Bugaj et al., 2015).

The success of this family of new optically sensitive protein-domain techniques has led to the development of a fairly robust phytochrome toolkit. These light-sensing domains found in plants, fungi, and bacteria are sensitive to longer wavelengths of light (650–760 nm), which improve compatibility with in vivo experiments due to increased tissue penetrance and decreased phototoxicity. Similar to the cryptochromes, the phytochrome system consists of a pair of heterologous subunits, phytochrome B (PhyB) and PIF6, that display light-dependent dimerization (Ni et al., 1999, p. 3). Although these tools have be used to control a wide variety of intracellular signaling cascades, a caveat to the plant-based phytochromes is that they require phycocyanobilin as a chromophore cofactor, which is not produced by mammalian cells. This has led to the development of bacterial phytochrome BphP1 and its partner PpsR2, which use biliverdin as a chromophore, a compound naturally produced in mammalian cells (Kaberniuk et al., 2016).

### B. Optogenetic Control of G Protein Activity

G proteins are the canonical effectors that couple GPCRs to a variety of intracellular signaling cascades. The receptors themselves act as guanine nucleotide exchange factors (GEFs), which drive release of bound GDP from the G proteins, which is then replaced with GTP. This nucleotide swap induces the dissociation of the *α* and *βγ* subunits, which each couple to unique effectors. Termination of G protein signaling occurs when the GTP in the alpha subunit is hydrolyzed to GDP, an event catalyzed by regulators of G protein signaling (RGS) proteins, leading to reconstitution of the inactive *αβγ* heterotrimer (Ross and Wilkie, 2000; Preininger et al., 2013). Multiple phases of the G protein activation/inactivation cycle have been probed using optogenetic strategies.

RGS proteins are one example in which optical methods for G protein control have been used. RGS proteins act as GTPase-activating proteins (GAPs), which catalyze the hydrolysis of GTP in G*α* subunits. These molecules have the ability to quickly curtail canonical G protein signaling. Early studies demonstrating the feasibility of optical approaches for manipulating RGS signaling used the CRY2-C1BN system to generate a CRY2-RGS4 fusion protein (O’Neill and Gautam, 2014). The RGS domain of the fusion lacked a key N-terminal membrane targeting domain, preventing it from expressing in its native pattern, proximal to membrane GPCRs. Using a membrane-bound C1BN subunit, the researchers were able to induce light-dependent translocation of this fusion to the plasma membrane. This approach was used to demonstrate that CXCR4 activation–induced G*βγ* translocation to intracellular compartments could be spatiotemporally reversed by blue light activation of RGS4 signaling. They further demonstrate that establishing an intracellular gradient of G protein signaling using CXCR4 activation in tandem with spatially restricted RGS activation was sufficient to promote cell migration in the opposing direction to the light illumination in macrophages.

One limitation to the CRY2-RGS4 technique is that RGS4 catalyzes nucleotide exchange from both G_i/o_ and G_q_ proteins. A subsequent study circumvented this caveat by engineering a CRY2 dimer with RGS2, which shows selectivity for the G_q_ subunit (Hannanta-Anan and Chow, 2018). A common consequence of G_q_ activation is an increase in intracellular calcium levels through positive modulation of voltage-gated calcium channels (VGCCs). Using a similar technique to prevent plasma membrane localization of RGS2 in the dark, they demonstrated that light-catalyzed translocation of the CRY2-RGS2 fusion to membrane-bound C1B1 acceptor resulted in reduced intracellular calcium through inhibition of the G_q_ signal transduction pathway.

These above approaches to drive RGS activity are useful for investigating the necessity of intact G protein signaling but do not directly assess the sufficiency of these pathways. To address these limitations, a study by Garcia-Marcos et al. (2020) set out to develop an optogenetic platform to directly drive G protein activity. Using a LOV2-based sequestration system, the authors fused a constitutive G*α*-binding and activating (GBA) motif to the C terminus of LOV2 (Garcia-Marcos et al., 2020). Using this approach, they demonstrated that 460–485 nm light irradiation was sufficient to induce G protein activation in both yeast and HEK293 cells.

Although optogenetic actuators of RGS and the heterotrimeric G protein complex have been useful in understanding the effects of manipulating broad G protein–signaling dynamics, they will necessarily modulate both the G*α* and G*βγ* effector arms. Hence, it does not attain the pathway specificity necessary to independently and directly assess the function of these disparate signaling cascades. Recently, other tools have been developed to create optically activatable G protein subunits. Using a PhyB-PIF6 based dimerization scheme, Sato and colleagues generated constitutively active G_q-_ and G_s_-PhyB chimeras that lacked membrane-targeting sequences, preventing them from accessing membrane-bound secondary messengers (Yu et al., 2016). After light-induced translocation of this protein to membrane-bound PIF6, the authors demonstrated increases in intracellular calcium and cAMP in the case of the G_q_ and G_s_ variants, respectively.

The feasibility of this approach for controlling individual G*α* protein subunits led to the application of these techniques for manipulating G*βγ* subunits. Similar to earlier studies using photoswitchable RGS proteins, initial attempts at controlling G*βγ* were made by altering activity of G protein–coupled receptor kinase 2 (GRK2). In addition to phosphorylating active GPCRs, GRK2 contains a pleckstrin homology domain in its C terminus, enabling it to directly interact with the G*βγ* subunit. This domain has been shown to be sufficient for sequestration of free G*βγ* subunits in vitro and was thus selected for generation of a photoswitchable tool for blockade of G*βγ* signaling. Importantly, because G*βγ* has a higher affinity for GDP-bound G*α* than GRK, the activation cycle of the G protein remains intact, as does G*α* signaling. A study by Gautam and colleagues demonstrated the feasibility of this approach using a chemoattractant to promote cell migration in a microphage-like cell line (O’Neill and Gautam, 2014). Here, a similar CRY2-C1BN system was implemented using a CRY2-GRKct fusion. They found that after global application of a chemoattractant, localized light stimulation on one side of the cell led to development of a phosphatidylinositol (3,4,5)-triphosphate (PIP_3_) gradient and cell migration toward the unstimulated side of the cell, demonstrating localized G*βγ* sequestration. Although these elegant studies demonstrate the ability to manipulate G*βγ* signaling using light, this system will uniformly inhibit all G*βγ* subtypes, limiting the selectivity of the technique. Mammals express 5 G*β* and 12 G*γ* subtypes, resulting in a myriad of possible combinations with different physiologic functions. In the future, it will be crucial to design tools to specifically target unique *β*-*γ* combinations to gain a more granular understanding of the varying signal transduction cascades that can be mediated by these proteins in a spatiotemporally precise manner.

### C. Optogenetic Control of Arrestin Signaling

Arrestins were initially characterized as intracellular molecules that drove cessation of GPCR signaling, either through steric hinderance of heterotrimeric G protein recruitment or by driving receptor internalization via clathrin-coated pits. However, decades of subsequent research demonstrate that arrestins represent a unique effector arm of GPCR signaling, driving a distinct cascade of intracellular signaling events (Pierce and Lefkowitz, 2001; Bruchas and Chavkin, 2010; Al-Hasani and Bruchas, 2011; Wingler and Lefkowitz, 2020). Arrestins are also capable of acting as scaffolds for a variety of kinases, including members of the mitogen-activated protein kinase (MAPK) family. As such, multiple tools and techniques have been devised to specifically address the physiologic function of this effector arm. Many of the chemical and genetic tools previously developed lack the temporal or spatial resolution needed to recapitulate the endogenous kinetics or control specific arrestin signaling networks. The development of optogenetic techniques to modulate arrestin activity has aided in ameliorating many of these issues.

One technique that has been used to probe the functional role of arrestin signaling cascade was creation of a chimeric optogenetically activatable *β*_2_-AR that displayed biased arrestin signaling through mutation of key residues involved in G protein recruitment (Siuda et al., 2015b, 2016). One drawback to this approach is that a novel mutant would have to be generated for each receptor to understand the interactions between that subtype and the arrestin signaling pathway. In many cases, we do not know how a particular GPCR subtype interacts with arrestin nor its time course or ultimate signaling output. Another caveat is that in some cases, arrestin signaling is driven by G protein–dependent recruitment of GRKs to the GPCR, such as GRK2 (Pitcher et al., 1992; Tesmer et al., 2005; Stoeber et al., 2020; Xiang et al., 2022). Hence, arrestin signaling driven by GRK2 phosphorylation of the GPCR will not be able to be assessed using the chimeric opsin approach, in which the C terminus has mutated GRK phosphorylation sites. Therefore, it would be useful to generate systems whereby arrestin signaling can be independently manipulated without the need for concurrent GPCR activation. This would provide two important avenues for exploring GPCR-mediated arrestin signaling in real time alongside of GPCR-independent arrestin signaling, which has been more recently demonstrated (Shukla et al., 2008; Chen et al., 2017; Gurevich and Gurevich, 2018; Haider et al., 2022; Kleist et al., 2022).

Alongside of the optogenetic approaches to manipulate G protein signaling, a CRY2-C1BN scheme has been used to assess the functional role of particular arrestin-mediated events. Two primary approaches have been developed that both use a CRY2-arrestin fusion protein. These techniques then entail generating either a GPCR-C1BN fusion or a plasma membrane–targeted C1BN, depending on the specific question being assessed. A recent study by using the former approach examined the effects of optogenetic recruitment of *β*-arrestin to the *β*_2_AR (Takenouchi et al., 2018). After blue light photostimulation, they observed rapid internalization of the receptor into clathrin-coated pits. Cessation of light irradiation induced a gradual reintroduction of these receptors back into the cell membrane. Although these studies demonstrated photoswitchable control of arrestin-dependent receptor internalization, these manipulations did not alter MAPK phosphorylation nor degradation of cAMP. Together, these results suggest that novel strategies must be developed to specifically assess the contribution of arrestin recruitment to activation of downstream kinase and second messenger signaling cascades (Haider et al., 2022).

### D. Optical Control of Second Messenger Systems

Second messengers are ubiquitous signaling molecules that propagate or amplify an incoming “first message” (i.e., ligand binding to a receptor) to a host of intracellular effectors. Second messengers of GPCR signaling include but are not limited to PIP_3_, IP_3_, cAMP, Ca^2+^, and diacylglycerol (DAG). Therefore, to gain a more precise understanding of the consequences of GPCR activation, it would be quite powerful to be able to individually manipulate these propagated second messenger signals with independent spatiotemporal specificity. Multiple optical tools have since been developed to accomplish this goal.

cAMP is a predominant second messenger of the G_s_ effector arm, and its production is regulated through GPCR-dependent adenylyl cyclase (AC) activity (Rodbell, 1997). Interestingly, a wealth of in-depth basic science studies revealed that a light-activatable AC occurs naturally in the bacterium *Beggiatoa* (Linder and Schultz, 2003). This particular isoform of AC is covalently linked to a BLUF domain, imbuing it with light-dependent activity. Heterologous expression of this protein in mouse hippocampal pyramidal neurons induced robust light-dependent depolarizing currents through cAMP-gated ion channels and was able to modulate grooming behavior in freely moving *Drosophila* (Stierl et al., 2011). A subsequent tool for photomanipulation of cAMP levels was developed from a mutated rhodopsin-guanylyl cyclase from *Catenaria anguillulae* (Scheib et al., 2018). By selective mutation of key residues in its nucleotide binding pocket, the authors were able to convert this molecule into a light-sensitive AC. This green light–sensitive protein offered far better reversal kinetics than the bacterial AC and was similarly compatible with mammalian systems. cAMP levels are also tightly regulated by phosphodiesterases (PDEs), which convert this molecule to AMP to curtail the cAMP signaling cascade. New approaches have also been developed to regulate cAMP levels using photosensitive AC and PDE in tandem, with each protein having unique spectral selectivity (Gasser et al., 2014; Stabel et al., 2019). However, it should be noted that similar to optical tools for modulating GPCR activity, it is unclear how faithfully light-sensitive ACs recapitulate native GPCR-elicited cAMP cascades. Under physiologic conditions, the vast majority of cAMP is not freely diffusing and is thought to be sequestered in cAMP binding sites, rendering it immobile (Bock et al., 2020). Furthermore, GPCRs and PDEs create cAMP nanodomains, which are crucial for selective propagation of the cAMP signal (Anton et al., 2022). Overexpression of light-sensitive ACs may drive unintended or nonspecific cAMP signaling that does not entirely recapitulate endogenous function of the native signaling cascade. Lastly, it should be noted that some of these tools similarly exhibit some basal activity in the dark, adding a further caveat to their implementation (Gasser et al., 2014).

G_q_ proteins exert their primary physiologic effects via activation of phospholipase C (PLC), which cleaves membrane-bound phosphatidylinositol biphosphate (PIP_2_) to generate soluble IP_3_ and membrane-bound DAG (Kostenis et al., 2020). This activity in turn opens calcium stores, often engaging various types of cell type–dependent release machinery, cellular activity, or cell polarity. Given the small size of these second messengers, they have typically been combined with photopharmacology approaches to manipulate their activity in a spatiotemporally specific manner. Photoswitchable DAG analogs have been created using the aforementioned azobenzene moiety to render it sensitive to light illumination (Leinders-Zufall et al., 2018). This offers the ability to assess the function of DAG, independent of IP_3_, which would not have been possible using chimeric opsin approaches. These experiments demonstrated light-dependent gating of transient receptor potential (TRP) channels through the use of a photoswitchable DAG. Despite the limited use of light-sensing protein domains for directly manipulating these small second messengers, optical control of heterotrimeric G proteins or kinases can offer indirect control over second messenger signaling cascades. For example, a study by Kakumoto and Nakata (2013) using a photoactivatable phosphoinositide 3-kinase (PI_3_K) demonstrated optical control over PIP_3_ levels. Several subsequent studies have built upon this approach by expanding the suite of optical tools to modulate the activity of kinases and other intracellular signaling proteins.

### E. Optogenetic Control of GTPase and Kinase Activity

Ras GTPases constitute a large family of signal transduction molecules implicated in a vast array of cellular processes. They are integral downstream signaling molecules of GPCRs, driving activity of a number of intracellular effectors such as the MAPKs. Ras proteins have both structural and functional homology to canonical G*α* subunits of heterotrimeric G proteins in that they act as molecular switches and have intrinsic GTPase activity (Reiner and Lundquist, 2018). Soon after the development of the optogenetics toolkit, similar engineering methods were applied to GTPases to exert control of their activity with enhanced spatial and temporal resolution. Using the *Arabidopsis thaliana* phytochrome system, Voigt and colleagues engineered a light-activatable GEF for Rho, a member of the Ras superfamily (Levskaya et al., 2009). Their system used a plasma membrane–anchored phytochrome B (PhyB) and a fusion protein of its binding partner photochrome interaction factor 3 (PIF3) and the catalytic domain of the guanine nucleotide exchange factor (GEF Tiam. Red light irradiation induced translocation of this GEF to the cell membrane, where it could catalyze the exchange of GDP to GTP in membrane-associated Rho molecules. They demonstrated this translocation-regulated organization of the actin cytoskeleton, causing pronounced lamellipodial phenotypes. Given the specificity of GEFs for unique Ras family members, this technique provides for the development of a host of tools of optogenetic modulation of specific GTPases (Karunarathne et al., 2015; Zhang and Cui, 2015).

One of the major signaling cascades downstream of ligand-induced GPCR activation is the MAPK cascade. These molecules form multitiered signaling complexes, often through scaffolding proteins such as arrestins (Raman et al., 2007; Gurevich and Gurevich, 2018). Sequential activation of these kinases generates a highly regulated communication pathway from activated membrane receptors to transcription factors in the nucleus. Canonical MAPK signaling pathways typically begin with activation of the MAP3K, which then phosphorylates a MAP2K, which in turn phosphorylates a MAP1K. Recently, an AsLOV2 strategy was devised to allow optogenetic control over the MAP1Ks, c-Jun N-terminal kinase (JNK), and p38 MAPK (Melero-Fernandez de Mera et al., 2017). In this system, a small JNK or p38 inhibitory peptide was fused to a mutant AsLOV2 platform. In the dark, this peptide was fused in such a way that it was unable to interact with these MAPKs. However, after a light-induced conformational change and unwinding of the linking J*α* helix, the peptide was capable of binding to the active site of these MAPKs, thus inhibiting their activity. A complementary approach was developed to inhibit upstream MAP2Ks (MEK1), utilizing the dimeric protein pdDronpa (Zhou et al., 2017). This protein displays cyan light–driven dissociation into its component monomers, which can then be reassociated with violet light illumination. By fusing this dimer to specific MEK1 sites that sterically occluded the catalytic domain, they were able to demonstrate photoreversible control of MEK1 signaling.

## V. The Future of Optopharmacology

Almost half a century of research has investigated the utility of light to control pharmacological and biochemical processes. Starting with early ligand uncaging experiments, the rapid temporal kinetics of optical tools for manipulating receptor signaling were quickly realized. The molecular biology revolution spurred the development of dozens of unique approaches to imbue ligands, receptors, or entire neurons with light-sensitive moieties. Despite these advances, the implementation of these tools in vivo has been markedly slower due to several technological and experimental hurdles that need resolution.

### A. Barriers To Address

In comparison with the Opto-GPCR toolkit, optogenetic techniques for controlling intracellular signaling cascades have been implemented in intact neural systems more infrequently. There is also an argument to be made that these tools were primarily developed to assess questions related to cell biology rather than systems neuroscience; ultimately, fundamental cellular mechanisms play a crucial role in determining the activity and function of neurons. One possibility is that, as noted above, overexpression of intracellular signaling molecules that retain some basal dark-state activity could induce detrimental off-target effects that alter the function or even health of the neuron (which has amplified importance as there is comparatively little adult neurogenesis). Further refinement and tailoring of the specificity and sensitivity of these tools, as well as careful assessment of their expression in neural systems, will be necessary for them to be widely adopted for systems neuroscience applications.

A principal caveat that has slowed the implementation of many of GPCR-targeting tools is the fact that we still lack the ability to optically control native subtype-selective GPCR signaling in a genetic or cell type–specific manner. Conventional photopharmacology techniques such as ligand uncaging or photoswitchable ligands provide temporally precise localization to manipulate ligand-receptor interactions. However, for incorporation into modern systems neuropharmacology approaches, these benefits still remain insufficient for disentangling the functional role of GPCRs on specific neural populations.

An example would be an experiment in which one might attempt to investigate the role of endocannabinoid signaling in the medial prefrontal cortex (mPFC), making use of the previously developed photouncageable 2-arachidonylglycerol (2-AG) (Laguerre et al., 2019). After cannulation and fiber optic implantation into the mPFC, this tool would allow you temporally specific control of 2-AG-CB1 signaling within one specific brain region. However, there are at least half a dozen afferent projections in mPFC that express the CB1 receptor presynaptically as well as numerous local inhibitory neuron populations and intralaminar excitatory projections. Thus, it would be challenging to discern the behavioral effects of photouncageable 2-AG signaling, as they could be mediated by any one of several different CB1-expressing neural populations within the mPFC.

A work-around would be to devise a tethered photoswitchable ligand strategy in which the CB1 receptors in the region of interest (e.g., in the ventral hippocampus) express the necessary mutations on the N terminus to allow for cysteine-maleimide conjugation so that the ligand can bind to the receptor. However, this would either necessitate the generation of a knockin mouse or heterologous expression of the mutant receptor using a viral method. In the latter case, heterologous expression can yield several unintended effects and will not necessarily recapitulate native receptor expression or function. This same pitfall applies to remotely tethered photoswitchable ligands and even an engineered Opto-CB1 based on the Opto-XR platform.

### B. Tools Needed

For in vivo systems, we ultimately need to gain optogenetic control over native receptors in a cell-type specific manner. Over the last five years, significant progress has been made toward this goal. A seminal 2017 paper by Tadross and colleagues described the generation of a novel pharmacological tool called drugs acutely restricted by tethering (DART) (Shields et al., 2017). In this approach, rather than genetically modifying the endogenous receptor, they drove cell type–specific expression of a cell membrane–anchored protein called HaloTag, which serves as a binding site for a chemical moiety called a HaloTag ligand (HTL). This HTL was then conjugated by a long flexible linker to a ligand for a native receptor, in this case the AMPA receptor. Importantly, the compound was synthesized in such a way that in its freely diffusing state, it had modest efficacy for the AMPA receptor. After attachment to the HaloTag and restriction to the cell membrane, the compound’s potency increased by several orders of magnitude, allowing for selective manipulation of an endogenous receptor with cell-type specificity.

One drawback to this approach is the inherent slower kinetics of conventional pharmacology approaches as well as residual nonselective agonist activity at off-target sites. By combining these drug tethering approaches with photopharmacology techniques, many of the methodological concerns with each respective tool could potentially be ameliorated. A study by Isacoff and colleagues recently pioneered an adapted version of this approach, using in this case a genetically driven SNAP-tag (analogous to a HaloTag), which served as the binding site for its cognate ligand benzylguanine (BG) (Donthamsetti et al., 2019). BG was connected by a long flexible linker to a photoswitchable azobenzene group, which itself was connected to a glutamate molecule. Addition of the azobenzene group allowed them to gain photoswitchable control over mGluR_2_ signaling, specifically in neurons that expressed the SNAP-tag. Although not characterized in vivo, this approach provides an important proof of concept for genetically targetable photoswitchable control over native GPCR populations. Given the comparative ease of synthesis of the tethered photoswitchable ligands compared with generation of knockin mice or chimeric GPCRs, this technique represents a crucial technical advancement for the investigation of functionally specific populations of natively expressed GPCRs in vivo. Other groups have implemented this orthogonal tethering approach to probe dopamine receptor (Donthamsetti et al., 2021b) and GPR55 signaling (Tobias et al., 2021).

Complementary approaches using nanobody-conjugated photoswitchable compounds are being developed to improve the efficiency and applicability of these techniques. A study by Farrants et al. (2018) demonstrated the feasibility of this approach for manipulating mGluR_2_ activity. This approach benefits from the fact that nanobodies can be easily tailored to bind motifs of interest (N termini of receptors, GFP tags, etc.) and they can either be directly administered or be genetically encoded. In the future, one can imagine an alternative system in which newly developed CRISPR/Cas9 mutagenesis techniques could be harnessed to induce specific point mutations on a receptor of interest, allowing for direct conjugation of a tethered photoswitchable ligand (Hunker et al., 2020). This could enable cell type–specific targeting of a pharmacological compound with incredibly tight temporal control over receptor signaling.

One possible reason for a lack of widespread adoption of these photopharmacology techniques in vivo is that many of these tethered photoswitchable ligands are too large or are otherwise unable to cross the blood-brain barrier (BBB). Therefore, in vivo experiments would require the coimplantation of both a fiber optic and a drug cannula, which must be connected to a laser power source and an infusion pump, respectively. Although certainly within the realm of feasibility, these experiments are complex and often prove to have detrimental effects on mouse behavior due to the number of distinct components that must be fastened to the animal’s head. Beginning in the early 2010s, work by the Rogers and Bruchas laboratories began developing wireless single component integrated devices for simultaneous drug delivery and optical stimulation (Jeong et al., 2015; Shin et al., 2017; Zhang et al., 2019). These “optofluidic” devices contained a lightweight battery-powered infrared wireless module for powering both the micro-LEDs and drug infusion system, which consisted of drug reservoirs and thermally expandable layers to push the drug into the brain. They demonstrated that real-time place preference driven by stimulation of ventral tegmental area (VTA)-NAc terminals could be blocked by local infusion of the D1 antagonist SCH23390 into the NAc. These devices would prove be a useful platform for in vivo photopharmacology experiments, owing to their small size and ability to perform drug infusions and optical stimulation concurrently.

Another method of delivering these compounds into the brain would be via Trojan horse or nanoparticle-mediated delivery systems. As mentioned above, the majority of photopharmacology compounds are either too large or do not contain the right chemical properties to allow for transport across the BBB. Trojan horse methodologies rely on the conjugation of the drug of interest to a molecule or peptide (such as transferrin) that binds to specific receptors on the endothelial lining of capillaries that make up the BBB, allowing for transport of the drug-peptide conjugation into the brain (Pardridge, 2006). One drawback to this technique is the large size of this molecule, which could interfere with its biologic activity at the receptor of interest unless a strategy is devised to cleave the drug-peptide conjugation upon transport into the brain. A separate strategy would be to use novel nanoparticle or liposomal delivery systems that would allow for transport of the unmodified drug into the central nervous system (Vieira and Gamarra, 2016). Liposomal preparations have already shown promise in transporting large biomolecules across the blood-brain barrier in clinical trials for the treatment of Alzheimer’s and Parkinson’s diseases. Given that the drugs packaged into the liposomes can be more or less unmodified, this may prove to be a compelling strategy for delivery of photopharmacological compounds across the BBB.

## VI. Concluding Remarks

Despite the fact that roughly 40% of FDA-approved pharmacological compounds target GPCRs, there remains a noted lack of understanding as to the physiologic and functional role of these receptors at the systems level across many biologic fields. The brain in particular represents a massively complex puzzle in which understanding GPCR signaling with cellular and even subcellular resolution will require the continued advancement of our techniques and methods for probing these unique biochemical signaling cascades. In this review, we attempted to outline several of the principal technological advancements that have occurred over the last half century that have significantly aided in our quest to better understand everything from molecular interactions between ligand and GPCR orthosteric binding domain all the way to transcriptional regulation in the nucleus. We hope that this summary of key optical methods will serve as a resource for future researchers interested in investigating the intricacies of GPCR signaling in vitro and in vivo across biologic systems.

## Abbreviations

A_2A_: adenosine 2A
*α*_1_AR: alpha-1 adrenergic receptor
AC: adenylyl cyclase
2-AG: 2-arachidonylglycerol
ASR: acoustic startle response
*β*_2_AR: beta-2 adrenergic receptor
BBB: blood-brain barrier
BLUF: blue light–using flavin adenine dinucleotide
CB1,2,: cannabinoid receptor 1,2
CXCR3,4: chemokine receptor 3,4
DAG: diacylglycerol
FDA: US Food and Drug Administration
FMN: flavin mononucleotide
GEF: guanine nucleotide exchange factor
GIRK: G protein–coupled inward rectifying potassium channel
GPCR: G protein–coupled receptor
GRK: G protein–coupled receptor kinase
5-HT_1A_, _2A_: serotonin receptor 1A, 2A
IP_3_: inositol triphosphate
LOV: light-oxygen voltage domain
mAChR: muscarinic acetylcholine receptor
MAPK: mitogen-activated protein kinase
mGluR: metabotropic glutamate receptor
mPFC: medial prefrontal cortex
NAc: nucleus accumbens
NAM: negative allosteric modulator
OPN3: encephalopsin
OPN5: neuropsin
PDE: phosphodiesterase
PhyB: phytochrome B
PIP_3_: phosphatidylinositol (3,4,5)-triphosphate
PLC: phospholipase C
PPO: parapinopsin
THC: Δ-9 tetrahydrocannabinol
